# REPLY BY THE AUTHORS: Re: Antibiotic prophylaxis prior to urodynamic study in patients with traumatic spinal cord injury. Is there an indication?

**DOI:** 10.1590/S1677-5538.IBJU.2019.0193.1

**Published:** 2019-09-02

**Authors:** Marcello Torres da Silva, André Luis Barboza, Maria Malen Pijoán, Paulo Sergio Siebra Beraldo

**Affiliations:** 1 Serviço de Urologia, Rede Sarah de Hospitais de Reabilitação, São Luís, MA, Brasil;; 2 Serviço de Urologia, Rede Sarah de Hospitais de Reabilitação, Brasília, DF, Brasil;; 3 Instituto Universitario Italiano de Rosario - Ciências Biomédicas, Rosario, Santa Fe, Argentina;; 4 Serviço de Lesão Medular, Rede Sarah de Hospitais de Reabilitação, Brasília, DF, Brasil

We thank you for your comments on our latest publication (1). Indeed, what has motivated us the most to carry out this investigation was the lack of evidence concerning the usage of antimicrobials prior to urodynamic studies in spinal cord injured patients (2). An unexpected result for us was the greater incidence of urinary tract infection upon patients with injuries at T6 or above, regardlessly of the use of antimicrobials prior to the procedure.

At this exact moment we are working on a new publication that details the incidence of urinary tract infection specifically amongst the 379 spinal cord injured patients with injuries at T6 or above, now considering the presence of autonomic dysreflexia and urodynamic parameters. The results are becoming clinically relevant.

Yours Sincerely,

Authors
